# The association between blood pressure variability and perihematomal edema after spontaneous intracerebral hemorrhage

**DOI:** 10.3389/fneur.2023.1114602

**Published:** 2023-03-16

**Authors:** Lotte Sondag, Axel Wolsink, Wilmar M. T. Jolink, Sabine Voigt, Marianne A. A. van Walderveen, Marieke J. H. Wermer, Catharina J. M. Klijn, Floris H. B. M. Schreuder

**Affiliations:** ^1^Department of Neurology, Donders Institute for Brain, Cognition and Behavior, Radboud University Medical Center, Nijmegen, Netherlands; ^2^Department of Neurology, Isala Hospital, Zwolle, Netherlands; ^3^Department of Neurology, Leiden University Medical Center, Leiden, Netherlands; ^4^Department of Radiology, Leiden University Medical Center, Leiden, Netherlands

**Keywords:** intracerebral hemorrhage, blood pressure variability, brain edema, perihematomal edema, blood pressure

## Abstract

**Background:**

Perihematomal edema (PHE) after spontaneous intracerebral hemorrhage (sICH) is associated with clinical deterioration, but the etiology of PHE development is only partly understood.

**Aims:**

We aimed to investigate the association between systemic blood pressure (BP) variability (BPV) and formation of PHE.

**Methods:**

From a multicenter prospective observational study, we selected patients with sICH who underwent 3T brain MRI within 21 days after sICH, and had at least 5 BP measurements available in the first week after sICH. Primary outcome was the association between coefficient of variation (CV) of systolic BP (SBP) and edema extension distance (EED) using multivariable linear regression, adjusting for age, sex, ICH volume and timing of the MRI. In addition, we investigated the associations of mean SBP, mean arterial pressure (MAP), their CVs with EED and absolute and relative PHE volume.

**Results:**

We included 92 patients (mean age 64 years; 74% men; median ICH volume 16.8 mL (IQR 6.6–36.0), median PHE volume 22.5 mL (IQR 10.2–41.4). Median time between symptom onset and MRI was 6 days (IQR 4–11), median number of BP measurements was 25 (IQR 18–30). Log-transformed CV of SBP was not associated with EED (B = 0.050, 95%-CI −0.186 to 0.286, *p* = 0.673). Furthermore, we found no association between mean SBP, mean and CV of MAP and EED, nor between mean SBP, mean MAP or their CVs and absolute or relative PHE.

**Discussion:**

Our results do not support a contributing role for BPV on PHE, suggesting mechanisms other than hydrostatic pressure such as inflammatory processes, may play a more important role.

## Introduction

Spontaneous intracerebral hemorrhage (sICH) is a devastating disease with a poor outcome (1, 2). Besides direct injury through compression and disruption of brain parenchyma (3), sICH elicits a secondary response that starts within hours after sICH and causes secondary brain injury (SBI) (4). Perihematomal edema (PHE), especially peak PHE, is considered an important imaging marker for SBI (4, 5), is associated with clinical deterioration and possibly also with poor functional outcome (5, 6). PHE grows fastest in the first 72 h, gradually increases up to 2–3 weeks (5, 7–10), after which it gradually decreases until it resolves after ~1–2 months (5).

Formation of PHE is driven by osmotic and hydrostatic pressure gradients [difference between intracranial pressure and systemic blood pressure (BP)], reflecting the blood brain barrier (BBB) permeability (5). Not only high systemic BP but also greater systemic BP variability (BPV) may increase the development of PHE in areas of impaired autoregulation (11).

Various measures of PHE are available, including absolute and relative PHE volume (aPHE and rPHE), and the edema extension distance (EED = radius of a sphere that equals the volume of PHE and ICH together – the radius of a sphere that equals ICH volume) (12). It is suggested that EED is independent of hematoma volume and therefore the most appropriate proxy measure of SBI (12). Targeting PHE could possibly improve outcome in patients with sICH. Treatment of high BP could theoretically ameliorate the formation of PHE, based on a decrease of the hydrostatic pressure gradient (13). Earlier work investigating the relationship between BP and formation of PHE showed conflicting results (4, 5, 14–21), and the effect of early treatment of elevated BP on functional outcome after sICH is controversial (22, 23). It was suggested that high BPV rather than high BP alone, contributes to the formation of PHE (15) and may worsen clinical outcome (11, 13, 17, 24–28). No previous study investigated the association between BPV and EED.

We aimed to investigate the association of BPV, measured by coefficient of variation (CV) of systolic BP (SBP) within the first week after sICH, with the formation of PHE measured by EED up to 21 days after sICH. Second we assessed the associations of mean SBP, CV of mean arterial pressure (MAP) and mean MAP within the first week after sICH with EED. Third we investigated the associations of these BP measurements with aPHE and rPHE volume.

## Materials and methods

This study was part of the Finding ETiology of spontaneous Cerebral Hemorrhage (FETCH) study, a prospective multicenter study in adults with CT-confirmed sICH that aimed to characterize underlying pathophysiological mechanisms. Patients were recruited between October 2013 and December 2018 in three Dutch hospitals [Radboudumc Nijmegen, University Medical Center Utrecht (UMCU) and Leiden University Medical Center (LUMC)]. The FETCH study was approved by the Medical Ethics Review Committee of the UMCU (NL43286.041.13; METC 13/270D). We obtained written informed consent from all patients. We conformed to ICMJE Recommendations.

### Patients

For the current study, we included patients who underwent 3 tesla (T) brain magnetic resonance imaging (MRI) within 21 days after symptom onset and had at least five measurements of BP registered within the first week after symptom onset. We collected baseline characteristics including date and time of symptom onset, Glasgow Coma Scale (GCS) score and National Institute of Health Stroke Scale (NIHSS) score on admission, systolic and diastolic BP measurements (SBP and DBP) during admission (preferably the following BP measurements were recorded: first BP on admission; every 4 h in the first 48 h; every 6 h from days 3 to 7), past medical history [hypertension, diabetes mellitus, hypercholesterolemia, coronary artery disease, previous ICH, ischemic stroke, transient ischemic attack (TIA) or atrial fibrillation/flutter], vascular risk factors (use of tobacco, and alcohol) and previous medication (oral anticoagulant drugs, platelet inhibitors, antihypertensive drugs, lipid lowering drugs). BP measurements were collected as part of routine care and were performed by inflatable cuff measurements at the brachial artery. Definitions can be found in the Supplementary material.

### Magnetic resonance imaging

3 T brain MRI [Siemens Healthineers, Erlangen, Germany (Radboudumc); Philips Healthcare, Best, The Netherlands (LUMC, UMCU)] was performed using a standard protocol, amongst which Fluid Attenuated Inversion Recovery (FLAIR), and blood sensitive sequences (T2^*^ in Radboudumc and UMCU; susceptibility-weighted imaging (SWI) in LUMC) (29).

One trained reader (AW) manually segmented ICH and PHE volumes on axial FLAIR sequences using ITK-SNAP 3.8 (http://www.itksnap.org/) (30), blinded for baseline characteristics and BP measurements. A second trained reader (LS) segmented ICH and PHE volumes of 10 patients, blinded for baseline characteristics, BP measurements and the results from the first reader, to determine inter-observer agreement using the intraclass correlation coefficient (ICC). The Supplementary material describes the segmentation protocol. We used Matlab 2014b to calculate absolute ICH volumes and PHE volumes based on the number of voxels and the voxel size in three directions. Intraventricular hemorrhage extension (IVH) was classified absent or present. We classified ICH location as deep (thalamus, basal ganglia), lobar (cerebral lobes) or infratentorial (brainstem or cerebellum).

### Outcomes

The primary outcome parameter was EED, calculated as $3\sqrt{\frac{aPHE+ICH\;volume}{\frac{4}{3}\pi }}-3\sqrt{(}\frac{ICH\;volume}{\frac{4}{3}\pi })$ (12). Secondary, we assessed aPHE, and rPHE ($\frac{\text{aaPHE}}{\text{ICH volume}}$).

### Data analyses

We calculated means, standard deviations (SD) and CV of SBP, and MAP (= $\left(\frac{SBP+\left(2^{*}DPB\right)}{3}\right))$ using all BP values from the first week after symptom onset. We verified our data for normal distribution, and in case of skewed distribution performed log-transformation. We primarily investigated the association between CV of SBP and EED using univariable linear regression. Next, multivariable linear regression was used to adjust for prespecified covariables age, sex, ICH-volume and interval between symptom onset and MRI, as these are known to influence the development of SBI (5, 31) and PHE (5, 10, 21, 32–34). We performed a sensitivity analysis including all BP measurements from the first 48 h after onset only. We performed a subgroup analysis for deep, lobar and infratentorial sICH separately. Second, we repeated univariable and multivariable linear regression, now assessing associations between mean SBP and mean and CV of MAP as independent determinants and EED. Third, we assessed the associations between mean and CV of SBP, mean and CV of MAP and aPHE or rPHE through univariable linear regression. We used SPSS version 25.0 (IBM Corp, Armonk, New York, USA) to perform all statistical analyses.

## Results

From the 153 patients in the FETCH study with a 3 T MRI, 94 patients had an MRI of good quality that was performed within 21 days. We finally included 92 patients (mean age 64 years) who had at least five BP measurements available within the first week. Baseline characteristics of included and excluded patients were similar (Supplementary Table 1). Baseline characteristics of included patients are summarized in Table 1. Median number of BP measurements was 25 (IQR 18, 30) in the first week, and 12 (IQR 9, 13) in the first 48 h. Median interval between symptom onset and MRI was 6 days (IQR 4, 11). In 18 patients (20%) MRI was performed within 72 h and in 55 patients (60%) within 7 days of symptom onset. Agreement in ICH and PHE volume measurements on MRI between the two readers was excellent, with an ICC of 0.95 for ICH volume and 0.99 for PHE volume. MRI findings are summarized in Table 3. In five patients, MRI showed more than 1 ICH. In these patients we segmented the largest ICH and its surrounding PHE.

**Table 1:** Baseline characteristics and MRI findings of included patients (*n* = 92).

**Characteristic**	
Age (years)	64 (15)
Men	68 (74%)
**Medical history**	
Hypertension	57 (62%)
Diabetes mellitus	15 (16%)
Hypercholesterolemia	32 (35%)
Myocardial infarction	4 (4%)
Coronary artery bypass grafting	2 (2%)
Previous ICH	3 (3%)
Previous ischemic stroke	8 (9%)
Previous TIA	6 (7%)
Atrial fibrillation/flutter	14 (15%)
**Smoking**	
Ever	53 (58%)
**Medication**	
Anticoagulants	20 (22%)
Platelet inhibitors	18 (20%)
Antihypertensive medication	45 (49%)
Statin	31 (34%)
Glasgow Coma Scale at admission, median (IQR)	15 (13, 15)
≤ 8	1 (1%)
9–12	19 (21%)
13–15	72 (78%)
NIHSS at admission, median (IQR)	6 (3, 12)
IVH at admission	32 (35%)
Mean SBP first week	149 (14)
Mean DBP first week	81 (10)
Mean arterial pressure (MAP) first week	103 (10)
CV SBP first week, median (IQR)	0.12 (0.04)
CV MAP first week, median (IQR)	0.12 (0.04)
ICH volume on admission CT, median (IQR)	17.1 (4.9, 31.0)
Surgical hematoma evacuation	3 (3%)
**MRI findings**	
Median time from symptom onset to MRI (IQR), days	6 (4, 11)
ICH location, *n* (%)
Lobar	42 (46%)
Deep	37 (40%)
Infratentorial	13 (14%)
ICH volume, median (IQR)	16.8 (6.6, 36.0)
aPHE (mL), median (IQR)	22.5 (10.2, 41.4)
rPHE (mL), mean (SD)	1.63 (1.13)
EED (cm), mean (SD)	0.53 (0.17)

Categorical variables are presented as number (%), and continuous variables as mean (standard deviation), unless otherwise specified.

**Figure 1:**
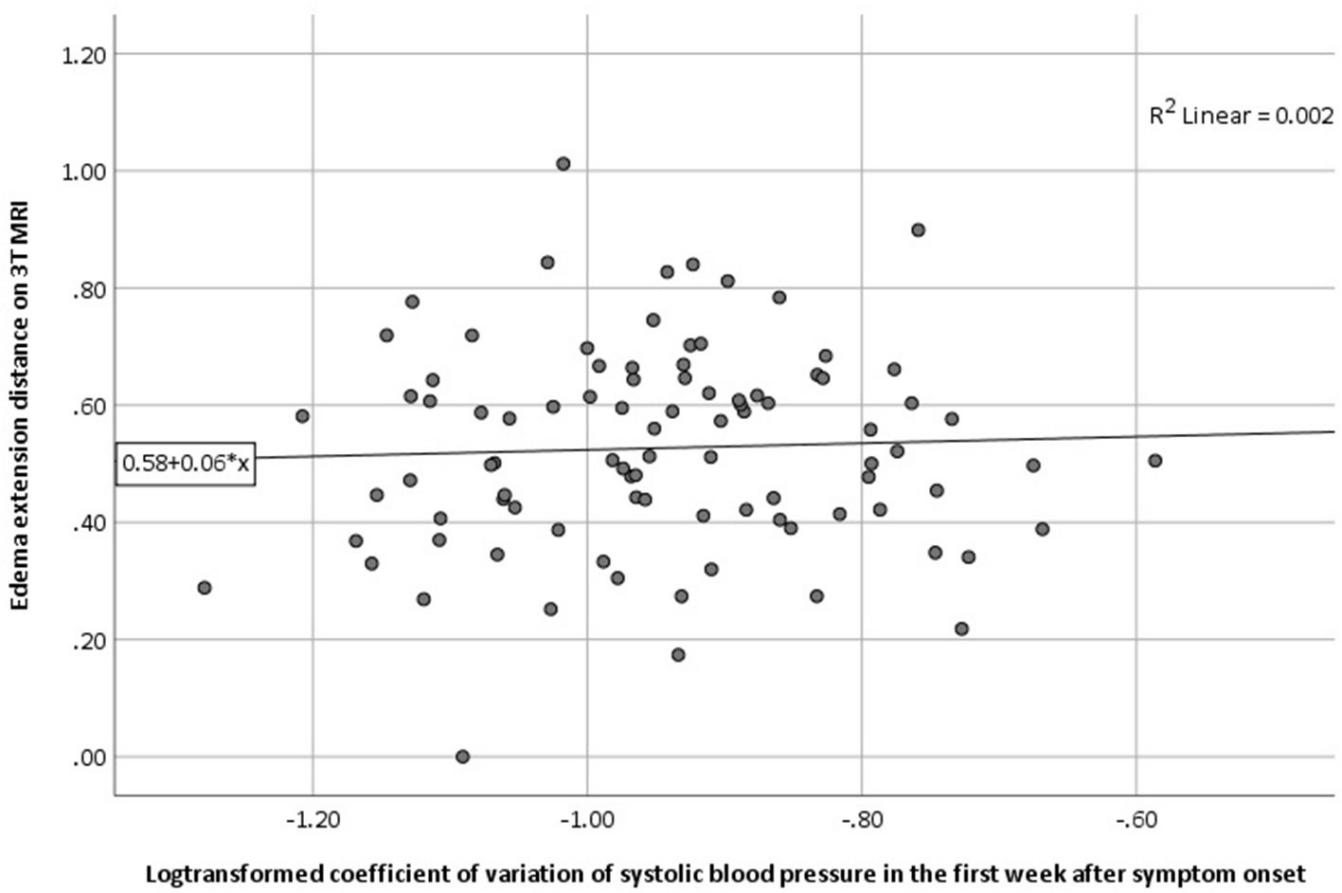
Association between (log-transformed) CV of SBP in the first week and the presence of PHE as measured by EED on 3T MRI (univariable regression analysis). 3T MRI, 3 tesla magnetic resonance imaging; CV, coefficient of variation; EED, edema extension distance; PHE, perihematomal edema; SBP, systolic blood pressure.

We found no association between log-transformed CV of SBP in the first week with EED in univariable regression analysis (B 0.056, 95% CI −0.002 to 0.003, *p* = 0.677; Table 2; Figure 1). This result remained unchanged after adjusting for age, sex, ICH volume on MRI and time between symptom onset and MRI (Table 3). Results remained unchanged in our sensitivity analysis including the BP measurements from the first 48 h after symptom onset only (Supplementary Table 2). In the subgroup analysis, both lobar, deep and infratentorial sICH did not show an association between CV of SBP in the first week and EED (Supplementary Table 3). Secondary analyses, we did not find an association between mean SBP, mean MAP or (log transformed) CV of MAP and EED in univariable and stepwise multivariable linear regression models (Tables 2, 3). Furthermore, we did not find an association between all BP measures and (log-transformed) aPHE or rPHE in univariable linear regression (Table 2).

**Table 2:** Univariable regression analyses for the influence of different BP measurements in the first week on PHE as measured by EED, aPHE, and rPHE on 3T MRI.

	**EED**	**Log-transformed aPHE**	**rPHE**
Log-transformed CV of SBP	B = 0.056 (−0.211 to 0.324)	B = −0.128 (−0.761 to 0.505)	B = 1.426 (−0.298 to 3.151)
	*P* = 0.677	*P* = 0.689	*P* = 0.104
	R^2^ = 0.002	R^2^ = 0.002	R^2^ = 0.029
Mean SBP	B < 0.001 (−0.002 to 0.003)	B < 0.001 (−0.007 to 0.006)	B = −0.002 (−0.019 to 0.015)
	*P* = 0.993	*P* = 0.917	P = 0.818
	R^2^ < 0.001	R^2^ < 0.001	R^2^ = 0.001
Mean MAP	B < 0.001 (−0.003 to 0.004)	B = 0.001 (−0.008 to 0.009)	B = 0.002 (−0.022 to 0.026)
	*P* = 0.844	*P* = 0.896	*P* = 0.868
	R^2^ < 0.001	R^2^ < 0.001	R^2^ < 0.001
Log-transformed CV of MAP	B = 0.031 (−0.216 to 0.330)	B = −0.069 (−0.712 to 0.575)	B = 0.853 (−0.923 to 2.629)
	*P* = 0.681	*P* = 0.832	*P* = 0.343
	R^2^ = 0.002	R^2^ = 0.001	R^2^ = 0.010

## Discussion

In patients with sICH, we found no association between variability of SBP (measured as CV of SBP) in the first week after symptom onset and EED on 3 T MRI within 21 days after symptom onset. Neither was there an association between mean SBP, mean MAP or CV of MAP and EED. Furthermore, we found no association between CV of SBP, mean SBP, mean MAP or CV of MAP and aPHE or rPHE.

BPV is associated with a higher risk of cardiovascular events and death due to cardiovascular diseases (28). Although the association between larger BPV and worse outcome after ischemic stroke and ICH has been established (11), few previous studies investigated the association between BPV and PHE (15, 17). A *post-hoc* analysis of the ATACH-2 trial (a randomized controlled trial investigating the effect of BP reduction < 140 vs. < 180 mmHg within 4.5 h after symptom onset) found a consistent association between different measures of BPV (SD, CV, average real variability, successive variation, residual SD) with clinical outcome in 913 participants, but not with absolute PHE growth on CT at 24 h as compared to baseline CT (17). A second study, that investigated 38 patients with sICH within 72 h after symptom onset, found an association of variability of MAP and minimal MAP in the first 72 h after symptom onset with rPHE on CT or MRI at 24 and 48–72 h, but not for mean MAP or maximal MAP (15). The absence of an association found in our study, could be partly explained by the difference in the modality and timing of PHE measurement. Instead of CT, we used MRI in all participants, which is a presumed to be a superior modality to delineate PHE (35). Additionally, most of our participants underwent MRI > 72 h after symptom onset, which corresponds better with the expected peak of PHE (5, 7, 9, 10). To take the influence of timing of MRI into account we have included this variable in the multivariable analysis, which did not alter our results. Furthermore, we measured EED, a more appropriate proxy measure of SBI compared to aPHE and rPHE (12). Another possible explanation for the different results is the absence of a standardized method to calculate BPV. Our study investigated mean and CV of SBP as well as mean and CV of MAP from all BP measurements within the first 7 days (and separately in the first 48 h). A previous study calculated hour-by-hour MAP-variance, mean MAP, maximal MAP and minimal MAP of all BP measurements performed, at least up until 72 h (15). The ATACH-2 trial calculated five measures of BPV (SD, CV, average real variability, successive variation, residual SD) from the highest and lowest SBP of every hour in the first 24 h, and the two highest and lowest SBP at days 2, 3, and 7 (17). However, in our sensitivity analyses including BP measurements from the first 48 h, results remained unchanged. Last, in our study we included a large proportion of patients that used oral anticoagulation (22 vs. 0% in the two previous studies), which may have influenced the development of PHE as thrombin (which is influenced by oral anticoagulation) can directly induce BBB-disruption and PHE development (36).

**Table 3:** Multivariable regression analyses for the influence of different BP measurements in the first week on the EED on 3T MRI.

**EED**	**Log-transformed CV of mean SBP**	**Mean SBP**	**Mean MAP**	**Log-transformed CV of MAP**
Model 1 (*n* = 92) [Age, sex]	B = 0.043 (−0.232 to 0.318)	B = 0.000 (−0.003 to 0.002)	B = 0.000 (−0.004 to 0.004)	B = 0.081 (−0.875 to 1.037)
	*P* = 0.756	*P* = 0.893	*P* = 0.937	*P* = 0.867
	aR^2^ = −0.011	aR^2^ = −0.012	aR^2^ = −0.012	aR^2^ = −0.012
Model 2 (*n* = 92) [Age, sex, (log-transformed) ICH volume at 3T MRI]	B = 0.090 (−0.150 to 0.330)	B = −0.001 (−0.003 to 0.001)	B = −0.001 (−0.004 to 0.002)	B = 0.306 (−0.618 to 1.051)
	*P* = 0.460	*P* = 0.448	*P* = 0.582	*P* = 0.608
	aR^2^ = 0.234	aR^2^ = 0.234	aR^2^ = 0.232	aR^2^ = 0.265
Model 3 (*n* = 84) [Age, sex, (log-transformed) ICH volume at baseline CT, time between symptom onset and 3T MRI]	B = 0.033 (−0.246 to 0.313)	B = 0.000 (−0.003 to 0.002)	B = 0.000 (−0.004 to 0.003)	B = −0.032 (−0.985 to 0.921)
	*P* = 0.812	*P* = 0.746	*P* = 0.850	P = 0.947
	aR^2^ = 0.078	aR^2^ = 0.072	aR^2^ = 0.071	aR^2^ = 0.070
Model 4 (*n* = 92) [Age, sex, (log-transformed) ICH volume at 3T MRI, time between symptom onset and 3T MRI]	B = 0.050 (−0.186 to 0.286)	B = −0.001 (−0.003 to 0.001)	B = −0.001 (−0.004 to 0.002)	B = 0.091 (−0.728 to 0.910)
	*P* = 0.673	P = 0.506	*P* = 0.600	*P* = 0.825
	aR^2^ = 0.273	aR^2^ = 0.275	aR^2^ = 0.274	aR^2^ = 0.272

Our study has several strengths. First, we selected patients from a prospective multicenter cohort study in which 3 T MRI was performed in all patients, irrespective of clinical deterioration. Second, we studied PHE on 3 T MRI up to 21 days, which is closer to represent peak PHE than studies that investigated PHE at 24 or 72 h. Third, we analyzed PHE on MRI scans, which is superior to CT for the investigation of PHE, which is illustrated by our excellent inter-observer reproducibility (37). Our study also has some limitations. The sample size was relatively small, which may have resulted in limited power. Two previous small studies reported discordant results regarding the association the between BPV and rPHE; these inconsistencies reflect small sample sizes and their associated imprecision and other biases (14, 15). Second, including only patients with MRI study has resulted in selection bias because severely affected patients were unable to undergo MRI or will have died before MRI could be performed. Third, time between symptom onset and MRI was not fixed, resulting in a variance between 1 and 19 days. Finally, BP measurements were not performed at standardized time intervals after symptom onset, and we included all BP measurements within the first week. Since exact timing of all BP measurements were not recorded, we were unable to include BP measurements prior to the MRI only. In addition, there was no clear and consistent protocol for the BP measurement itself. Differences in timing, equipment and operators can contribute to BPV. However, our sensitivity analysis using the BP measurements from the first 48 h only shows similar results. Although other studies showed an association between BPV and clinical outcome, our results do not support a role for BPV as a contributing factor for the development of PHE, suggesting that mechanisms other than hydrostatic pressure factors such as inflammatory processes, may play a more important role.

## Data availability statement

The raw data supporting the conclusions of this article will be made available by the authors, without undue reservation.

## Ethics statement

The studies involving human participants were reviewed and approved by Medical Ethics Review Committee of the University Medical Centre Utrecht, the Netherlands. The patients/participants provided their written informed consent to participate in this study.

## Author contributions

LS, FS, and CK contributed to the conception and design of the current study. CK, FS, MWa, MWe, and WJ contributed to the conception, design, and execution of the FETCH study. CK, FS, LS, MWa, MWe, SV, and WJ contributed to patient inclusions and data collection. LS and AW performed statistical analyses and wrote the draft of the article. All authors contributed to manuscript revision, read, and approved the submitted version.
