# Supplementary material for: The association between blood pressure variability and perihematomal edema after spontaneous intracerebral hemorrhage
Source: Front Neurol. 2023 Mar 16;14:1114602. doi: 10.3389/fneur.2023.1114602 (PMC10060834; doi:10.3389/fneur.2023.1114602)
Supplement: Supplementary file 1 [file Data_Sheet_1.docx]

Supplementary Material

**The association between blood pressure variability and perihematomal edema after spontaneous intracerebral hemorrhage**

**1 Supplementary data**

**Protocol for imaging analyses**

One trained reader (AW) manually segmented ICH and PHE volumes on axial FLAIR sequences using ITK-SNAP 3.8 (http://www.itksnap.org/),^1^ blinded for baseline characteristics and blood pressure measurements. A second trained reader (LS) segmented ICH and PHE volumes of 10 patients, blinded for baseline characteristics, blood pressure measurements and the results from the first reader, to determine inter-observer agreement using the intraclass correlation coefficient (ICC). Hemorrhage was defined as hyperintense on FLAIR sequences if the MRI was performed within 12 hours or beyond 8 days after symptom onset, and hypointense if MRI was performed between 12 hours and 8 days after symptom onset.^2^ If we were unsure if a hypointense or hyperintense region consisted of blood, we used T2* and SWI sequences to differentiate.^3^ The hyperintense region surrounding the hemorrhage on FLAIR sequences was segmented and classified as PHE.^4^ A distinction between PHE and white matter hyperintensities was made by visual comparison with the hyperintensities in the contralateral hemisphere, the location of the hyperintensity and subtle differences in intensity within the hyperintensities. In case of multiple hemorrhages, we segmented the largest hemorrhage and its surrounding edema on MRI.

**References**

1. Yushkevich PA, Piven J, Hazlett HC, et al. User-guided 3D active contour segmentation of anatomical structures: significantly improved efficiency and reliability. *Neuroimage*. 2006;31(3):1116-1128.

2. Kidwell CS, Wintermark M. Imaging of intracranial haemorrhage. *Lancet Neurol*. 2008;7(3):256-267.

3. Patel MR, Edelman RR, Warach S. Detection of hyperacute primary intraparenchymal hemorrhage by magnetic resonance imaging. *Stroke*. 1996;27(12):2321-2324.

4. Linfante I, Llinas RH, Caplan LR, Warach S. MRI features of intracerebral hemorrhage within 2 hours from symptom onset. *Stroke*. 1999;30(11):2263-2267.

**2 Supplementary data: Definitions of baseline characteristics**

Hypertension: systolic blood pressure >140mmHg or diastolic blood pressure >90mmHg in two separate measurements, the use of antihypertensive medication or evidence of left ventricular hypertrophy on the ECG.

Diabetes mellitus: known in medical history or two fasting measurement >7mmol/l.

Hypercholesterolemia: total cholesterol >6.2mmol/l or the use of lipid lowering medication.

**Supplementary Table 1. Baseline characteristics of included and excluded patients**

|  | **No MRI performed (n=51)** | **Other reason for exclusion (n=61)#** | **Included patients** |
| --- | --- | --- | --- |
| Age, median (IQR) | 69 (13) | 62 (14) | 64 (15) |
| Male sex, n(%) | 30 (59) | 43 (70.5) | 68 (74) |
| GCS, median (IQR) | 14 (12.75, 15) | 14 (13, 15) | 15 (13, 15) |
| NIHSS, median (IQR) | 6 (3, 14) | 4 (2, 7) | 6 (3, 12) |
| ICH volume on MRI, median (IQR)* | NA | 15.3 (6.3, 24.0) | 16.8 (6.6, 36.0) |
| ICH volume on baseline CT, median (IQR) | 25.3 (8.1, 29.3) | 16.9 (7.0, 33.4) | 17.1 (4.9, 31.0) |
| PHE volume on MRI*, median (IQR) | NA | 3.5(0, 12.8) | 22.5 (10.2, 41.4) |
| Days between symptom onset and MRI, median (IQR) | NA | 48 (32, 80) | 64 (15) |

* for patients with good quality MRI
# 53 patients underwent MRI after 21 days, 6 patients had incomplete or poor quality MRI and 2 patients had less than 5 BP measurements available.

**Supplementary Table 2. Multivariable regression analyses for the influence of CV of mean SBP in the first week and in the first 48 hours after symptom onset separately on EED on 3TMRI**

| **EED** | **Log-transformed CV of mean SBP  in the first week** | **Log-transformed  CV of mean SBP  in the first 48 hours** |
| --- | --- | --- |
| ***Univariable*** |  |  |
| EED | B 0.056  (CI -0.211-0.324) P=0.677 R^2^ = 0.002 | B -0.023  (CI -0.222-0.176)  P=0.815  R^2^ = 0.001 |
| ***Multivariable models*** |  |  |
| Model 1  (age, sex) | N=92  B 0.043  (CI -0.232-0.318)  P=0.756  aR^2^ = -0.011 | N=91  B – 0.029  (CI -0.234-0.176) p=0.780 aR^2^ -0.012 |
| Model 2  (age, sex, (log-transformed) ICH volume at baseline CT) | N=84  B 0.066  (CI -0.219-0.352)  P=0.645  aR^2^ = 0.035 | N=83  B -0.010  (CI -0.222-0.202)  p=0.926  aR^2^ 0.032 |
| Model 3  (age, sex, (log-transformed) ICH volume at 3TMRI) | N=92  B 0.090  (CI -0.150-0.330)  P=0.460  aR^2^ = 0.234 | N=91 B=0.058  (CI -0123-0.239) p=0.527 aR^2^ 0.237 |
| Model 4  (age, sex, (log-transformed) ICH volume at baseline CT, time between symptom onset and 3TMRI) | N=84  B 0.033  (CI -0.246-0.313)  P=0.812  aR^2^ = 0.078 | N=83 B= 0.014  (CI -0.222-0.193) p=0.890 aR^2^ 0.070 |
| Model 5  (age, sex, (log-transformed) ICH volume at 3TMRI, time between symptom onset and 3TMRI) | N=92  B 0.050  (CI -0.186-0.286)  P=0.673  aR^2^ = 0.273 | N=91 B= 0.055  (CI -0.122-0.231) p=0.540  aR^2^ 0.277 |

**Supplementary Table 3. Univariable regression analyses for the influence of CV of mean SBP in the first week after symptom onset separately on EED on 3TMRI in subgroups based on ICH location.**

| **EED** | **Lobar** | **Deep** | **Infratentorial** |
| --- | --- | --- | --- |
| ***Univariable*** |  |  |  |
| EED | B=-0.055 (CI -0.388- 0.278) p=0.740 | B=-0.142  (CI -0.546-0.262) p=0.480 | B=0.113  (CI -0.322-0.547) p=0.580 |
